# Bridging Pancreatic Amyloidosis and Neurodegeneration: The Emerging Role of Amylin in Diabetic Dementia

**DOI:** 10.3390/ijms26115021

**Published:** 2025-05-23

**Authors:** Gourav Shome, Ritwick Mondal, Shramana Deb, Jayanta Roy, Atin Kumar Mandal, Julián Benito-León

**Affiliations:** 1 Department of Biological Sciences, Bose Institute, Kolkata 700054, India; gshome007@gmail.com (G.S.); mandalak@jcbose.ac.in (A.K.M.); 2 Department of Neurology, Manipal Group of Hospitals Kolkata, Kolkata 700099, India; ritwickraw@gmail.com (R.M.); shramanadeb1039@gmail.com (S.D.); jroyneuro01@gmail.com (J.R.); 3 Department of Neurology, 12 de Octubre University Hospital, 28041 Madrid, Spain; 4 Group of Neurodegenerative Diseases, Hospital Universitario 12 de Octubre Research Institute (imas12), 28041 Madrid, Spain; 5 Network Center for Biomedical Research in Neurodegenerative Diseases (CIBERNED), 28029 Madrid, Spain; 6 Department of Medicine, Faculty of Medicine, Complutense University, 28040 Madrid, Spain

**Keywords:** Alzheimer’s disease, type 2 diabetes mellitus, dementia, amylin, amyloid-β, human islet amyloid polypeptide (human amylin), heat shock protein (HSP), proteostasis, aging

## Abstract

A hallmark of type 2 diabetes mellitus (T2DM) is the presence of abundant amyloid deposits composed of amyloid polypeptide (amylin) within the pancreatic islets of Langerhans. Given its high prevalence among diabetic individuals, human amylin fibrillization has long been considered a key pathogenic factor in T2DM. Co-secreted with insulin, amylin can misfold and aggregate, inducing β-cell toxicity, impairing insulin secretion, and accelerating disease progression. Emerging evidence also indicates that amylin accumulates in the brains of patients with Alzheimer’s disease, where it may interact with amyloid-β (Aβ) to promote neurodegeneration. Although the underlying mechanisms remain under investigation, amylin aggregates have been shown to disrupt mitochondrial function, trigger endoplasmic reticulum stress, and activate the NLRP3 inflammasome. Additionally, T2DM-associated cerebrovascular alterations may compound cognitive decline. This review, based on a comprehensive literature search across major biomedical databases up to January 2025, synthesizes current evidence on amylin as a molecular link between metabolic and neurodegenerative disorders. We highlight pancreatic β-cell amylin aggregation as a potential early marker of dementia risk in T2DM and examine its relationship with proteostasis-associated proteins. Finally, we discuss emerging diagnostic and therapeutic strategies targeting amylin pathology, offering new perspectives on preventing or delaying neurodegeneration in individuals with T2DM.

## 1. Introduction

Protein aggregation disorders are characterized by the misfolding of native proteins, leading to their pathological accumulation. A subset of these proteins adopts β-pleated sheet-rich conformations upon misfolding, forming amyloid aggregates—a hallmark of a group of disorders collectively termed amyloidosis [1,2]. In the context of type 2 diabetes mellitus (T2DM), islet amyloid deposits represent a prominent pathological feature, found in over 90% of affected individuals upon postmortem histopathological examination [3,4,5]. A strong correlation has been established between amylin aggregation and the progressive loss of pancreatic β-cell mass. In cases where substantial islet amyloid deposition is observed, there is often a concurrent increase in β-cell apoptosis [4].

Amylin, also known as islet amyloid polypeptide, is a 37-amino acid peptide hormone and the principal constituent of islet amyloid deposits in T2DM [6]. It is co-localized with insulin in the secretory granules of pancreatic β-cells and is co-secreted with insulin in response to β-cell stimulation [3]. Physiologically, amylin plays essential roles in glucose homeostasis by regulating gastric emptying, promoting satiety, suppressing glucagon secretion from pancreatic α-cells, and modulating insulin output [3,7,8,9,10,11]. In healthy individuals, insulin and mature amylin are secreted at a molar ratio of approximately 100: 1 (insulin: amylin). In T2DM, chronic hyperinsulinemia is typically accompanied by elevated plasma levels of amylin [3].

Beyond concentration, several factors influence the amyloidogenic potential of amylin, including the cellular microenvironment, proteostatic imbalance, oxidative stress, and genetic mutations. Among these, pH fluctuations have emerged as key modulators of amylin aggregation. Specifically, aggregation is more favorable at slightly basic pH compared to acidic conditions [10]. This observation aligns with the physiological transition that occurs when amylin is secreted from the acidic milieu of pancreatic secretory granules (pH~5.5) into the extracellular space (pH~7.4), potentially impairing its degradation and promoting its deposition as an islet amyloid [7].

A growing body of recent evidence supports a robust association between metabolic syndrome and late-onset dementias [12,13,14,15]. Notably, individuals with T2DM are estimated to have a two- to five-fold increased risk of developing Alzheimer’s disease (AD) and related dementias compared to non-diabetic individuals [12,13,14,15,16,17,18]. Furthermore, AD-associated neuropathologies—including extracellular amyloid-β (Aβ) plaques—may exacerbate cerebral insulin resistance, suggesting the presence of a bidirectional feedback loop in which amyloid accumulation and insulin signaling deficits reinforce one another [17,18,19,20]. This interplay may contribute to progressive neurodegeneration and cognitive decline.

In this review, we examine the potential role of amylin as a molecular bridge between metabolic dysregulation and neurodegeneration. Specifically, we explore how amylin-induced proteostatic stress might link pancreatic β-cell dysfunction in T2DM with the pathophysiological features of AD and related dementias.

## 2. Methods

We conducted a detailed and systematic literature review using several major databases, including PubMed/Medline, The Cochrane Library, and open-access platforms such as bioRxiv, MedRxiv, and preprint.org, covering the literature published up to January 2025.

The initial search strategy combined MeSH terms and keywords such as “islet amyloid polypeptide”, “amylin”, “Alzheimer’s disease”, and “amyloid-β”, retrieving 348 articles. A second search expanded the scope using terms such as “vascular dementia”, “diabetogenic stress”, “ER stress”, “arteriosclerotic dementia”, “metabolic syndrome”, “type 2 diabetes”, “dementia”, and “proteostasis dysfunction”, yielding an additional 276 articles. A third search focused on more specialized terms, including “heat shock proteins”, “amylin–Aβ interactions”, “amylin–endothelial interactions”, “β-cell dysfunction”, “β-cell apoptosis”, and “post-translational modification”, which generated 124 further results for in-depth review.

The search process was conducted collaboratively by GS, RM, and SD. RM, AKM, and JR meticulously screened abstracts. Full-text reviews of eligible articles were performed by AKM and JBL, with JR and JBL also assessing those articles lacking abstracts or containing insufficient preliminary information. All extracted findings were thoroughly reviewed and discussed by the entire research team, including RM, GS, SD, JR, AKM, and JBL, ensuring accuracy and consensus in article selection and data interpretation.

Of the 748 articles initially identified across all searches, 87 unique articles were retained after the removal of duplicates and full-text screening. The final synthesis prioritized studies that directly investigated amylin aggregation, cytotoxicity, cross-seeding with Aβ, or proteostasis-related mechanisms.

## 3. Results and Discussion

### 3.1. Molecular Insights into Amylin Aggregation and Proteostasis Impairment

Amylin exhibits species-specific aggregation propensities, with human amylin being highly amyloidogenic. Human amylin is prone to form β-sheet fibrils (similar in structure to Aβ), whereas rodent amylin carries proline substitutions at key positions and does not form amyloid fibrils [21,22,23]. Human amylin aggregates accumulate mainly extracellularly, though small amounts have been reported intracellularly in β-cells [24,25,26]. Emerging evidence indicates that human amylin exerts cytotoxic effects on β-cells, ranging from membrane disruption and reactive oxygen species production to the induction of apoptosis and inflammasome activation. However, the precise cellular pathways mediating amylin toxicity remain to be fully elucidated.

**Figure 1:**
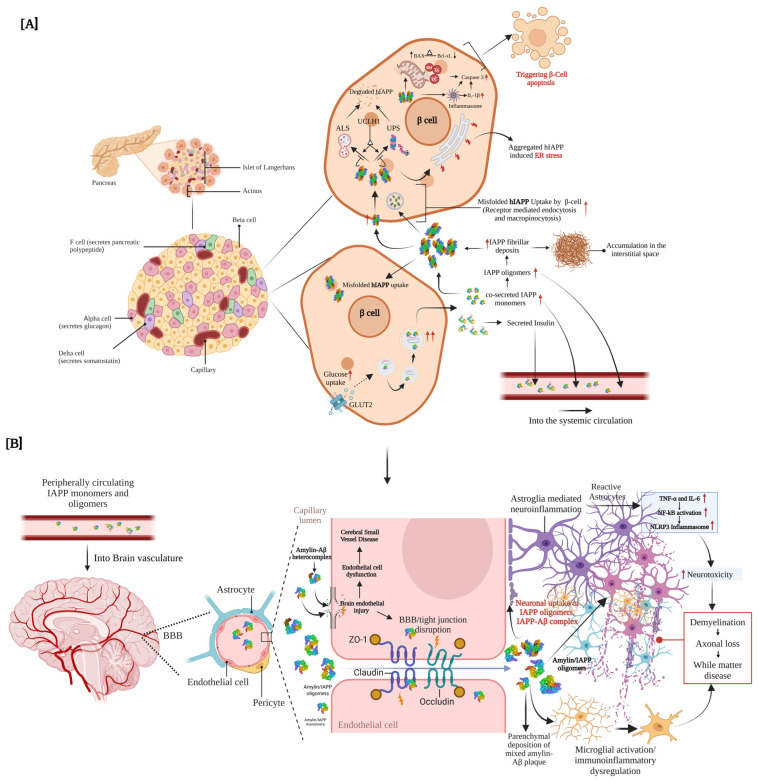
Pathological Roles of Islet Amyloid Polypeptide in Pancreatic β-Cells and Neurovascular Dysfunction in Type 2 Diabetes Mellitus (T2DM). (**A**) This schematic illustrates the pathophysiological processes triggered by human amylin (islet amyloid polypeptide, IAPP) in the pancreatic islets of Langerhans under diabetogenic conditions. In response to elevated glucose, amylin is co-secreted with insulin from pancreatic β-cells. However, under chronic metabolic stress, human amylin may misfold and aggregate into toxic oligomers and fibrils, which accumulate both intracellularly and extracellularly via receptor-mediated endocytosis and macropinocytosis. These aggregates induce endoplasmic reticulum (ER) stress and activate the unfolded protein response (UPR), disrupting proteostasis mechanisms such as the autophagy-lysosome system (ALS) and the ubiquitin-proteasome system (UPS) [32,45,47]. Persistent proteotoxic stress leads to mitochondrial dysfunction, an imbalance in pro- and anti-apoptotic signals (↑BAX, ↓Bcl-xL) [37], elevated reactive oxygen species (ROS) production, and enhanced secretion of proinflammatory cytokines such as interleukin-1β (IL-1β) [33,36]. These interlinked processes culminate in β-cell apoptosis. The resultant progressive β-cell loss exacerbates amylin aggregation and insulin dysregulation, establishing a self-perpetuating cycle that accelerates T2DM progression. (**B**) This panel depicts the downstream neurovascular effects of peripherally circulating amylin oligomers and fibrils. Amylin can cross the blood-brain barrier (BBB) and accumulate in cerebral tissue, where it may co-aggregate with amyloid-β (Aβ) to form heterocomplexes. These aggregates activate neuroinflammatory signaling cascades, including the NLRP3 inflammasome [33] and the IL-6 axis [36], contributing to BBB tight junction disruption, endothelial dysfunction, and astrocyte reactivity. Downstream effects include axonal degeneration, demyelination, white matter lesions, and microglial dysregulation, all of which are commonly implicated in diabetic dementia and Alzheimer’s disease. This figure highlights how peripheral metabolic dysfunction, initiated in pancreatic β-cells, contributes to central neurodegeneration.

Furthermore, consistent with early mechanistic observations, ex vivo studies have demonstrated that human amylin aggregates induce β-cell apoptosis, with deposits observed along the cell membrane [24]. Subsequent research has shifted the focus from fibrillar aggregates to soluble oligomeric forms of amylin as the principal toxic species. These oligomers have been shown to trigger β-cell apoptosis more effectively, a finding corroborated in both diabetic animal models and elderly human subjects [25]. Proposed mechanisms include endoplasmic reticulum (ER) stress and the disruption of key protein quality control pathways, such as the ubiquitin-proteasome system and the autophagy-lysosome system (Figure 1A).

In recent years, increasing attention has been paid to the cytotoxicity of oligomeric amylin species, which appear to be more toxic than mature fibrils [26,27,28,29,30,31,32,33,34,35]. Notably, ex vivo experiments demonstrated increased apoptosis in β-cells treated with freshly added human amylin peptides, compared to cells exposed to fibrillar aggregates [27]. Patients with T2DM have also been found to exhibit elevated levels of interleukin-1β (IL-1β), which amplifies the proinflammatory milieu and contributes to β-cell death [36]. Complementary cell culture studies revealed that oligomeric amylin activates the NLRP3 inflammasome, leading to IL-1β production and inflammation-driven apoptosis [33] (Figure 1B).

Further evidence from Yoo et al. [37], using INS-1E cells, showed that stable overexpression of human amylin promotes oligomer formation, which in turn induces both ER stress and oxidative stress. This stress response upregulates pro-apoptotic genes such as Bax and mTOR while concurrently downregulating Bcl-2 and genes encoding mitochondrial complexes I–IV, compared to control cells. These changes culminate in mitochondria-mediated apoptosis, reinforcing the notion that oligomeric amylin drives β-cell death through mitochondrial dysfunction (Figure 1A).

Studies on amylin clearance by the ubiquitin-proteasome system have yielded mixed results. Casas et al. [32]. reported that extracellular human amylin exposure reduced proteasomal activity more than amylin in a murine insulinoma cell line. Moreover, human amylin aggregation was associated with ER stress, whereas non-amyloidogenic amylin did not form aggregates and is often used as a control in proteostasis studies. However, other experiments found that adenoviral overexpression of human amylin or amylin in cells increased proteasomal activity, and isolated pancreatic islets from transgenic rats showed no significant difference in proteasome function between human amylin and amylin conditions [38]. These findings suggest that amylin does not invariably target ubiquitin-proteasome system pathways and that alternate clearance mechanisms may compensate in some contexts. (Figure 1A).

### 3.2. Human Amylin-Mediated Proteotoxic Stress: Impact on Ubiquitin–Proteasome and Autophagy–Lysosome Systems

Microarray studies have shown that treating β-cells with human amylin can downregulate key chaperones, including cytosolic Hsp90AA1/AB1 and ER chaperone Hsp90B1 [32,39]. Hsp90 is a chaperone that assists in 26S proteasome assembly and prevents oxidative inactivation of the 20S proteasome [40,41]. Thus, reduced Hsp90 levels could contribute to decreased proteasomal activity. Indeed, human amylin-treated mouse insulinoma (MIN6) cells, human amylin-transgenic rats, and human amylin-transduced INS 832/13 insulinoma cells all showed an accumulation of polyubiquitinated proteins compared to controls with amylin [32,42]. In contrast, another β-cell line, RIN-m5F, did not show a marked increase in ubiquitinated proteins upon human amylin treatment [43]. Notably, patients with T2DM have significantly lower levels of the deubiquitinating enzyme UCH-L1, along with decreased proteasome activity, resulting in the intracellular buildup of ubiquitinated proteins [42]. This finding raises the question of whether human amylin is degraded predominantly by the 20S proteasome (in a ubiquitin-independent manner) or also via the ubiquitin-dependent 26S proteasome.

In vitro studies indicate that ubiquitination of amylin is not required for 20S proteasomal recognition. However, immunofluorescence studies show strong co-localization of human amylin with ubiquitin in human amylin-treated cells and human islets, and intracellular human amylin can itself be ubiquitinated [43]. Furthermore, human amylin co-precipitated with components of the 26S proteasome (such as the 19S subunit Rpn8 and the 20S subunit PSMA4) and co-localized with chaperones Hsp70 and Hsp90 [43]. These results suggest that a ubiquitin-dependent pathway may also be involved in clearing human amylin. Consistently, proteasomes can degrade human amylin under both aggregation-prone and aggregation-inhibiting conditions, implying that human amylin does not need to form aggregates to be targeted by the proteasome [43,44].

Autophagy is another crucial route for protein quality control. One of the earliest studies linking amylin and autophagy showed that transfecting COS-1 cells (which lack endogenous amylin) with human amylin led to enhanced autophagosome formation compared to amylin transfection [45,46]. Similarly, INS-1 cells treated with human amylin, INS-1 cells transduced with human amylin, and islet β-cells from transgenic rats and mice all showed increased autophagosome accumulation compared to amylin control groups. [47,48]. Further, it was noted that even amylin-transduced INS 832/13 cells had more autophagosomes than non-transduced cells, though far fewer than human amylin-transduced cells. It remains unclear whether the observed autophagosome increase reflects an overall rise in autophagic flux or a backlog due to impaired lysosomal degradation.

Hence, to clarify autophagy’s role in degrading toxic human amylin, researchers have used pharmacological and genetic modulators. Inhibition of lysosomal proteases with pepstatin A markedly increased human amylin-induced apoptosis in β-cells, accompanied by higher intracellular amylin content in INS 832/13 cells and human islets [44,47]. Similarly, Atg7 knockout (autophagy-deficient) cells showed greater human amylin-induced apoptosis than rat amylin-treated cells [44,47,48]. Conversely, stimulating autophagy with rapamycin (an mTOR inhibitor) significantly reduced human amylin-induced apoptosis and lowered intracellular amylin levels. These changes were not due to altered amylin expression or secretion, and insulin content was unaffected, indicating that autophagy actively regulates amylin turnover. Indeed, rapamycin-induced autophagy appears to protect β-cells by facilitating amylin clearance [49,50]. Rivera et al. [47] reported that human amylin becomes polyubiquitinated in cells, potentially targeting it for p62-mediated autophagy. Ubiquitination could also mark proteins for 26S proteasomal degradation, so both pathways may converge on clearing amylin aggregates.

Different pathways may handle intracellular vs. extracellular amylin. In one study, exogenous human amylin or rat amylin was added to RIN-m5F cell cultures to model the uptake of circulating amylin. After uptake, amylin localized predominantly to the nucleus, with very little co-localizing to lysosomes [43]. In this context, inhibiting autophagy with pepstatin A did not significantly change intracellular amylin levels [43]. These observations underscore that cells employ multiple clearance mechanisms for amylin, and the predominant pathway may depend on how and where the protein is encountered.

Collectively, these findings underscore the complex proteostatic stress induced by misfolded amylin. Early-stage aggregation may serve as a biomarker for disease progression or for identifying individuals at risk of T2DM-associated cognitive decline. Disruption of either the proteasomal or autophagic pathways exacerbates amylin toxicity, highlighting the importance of preserving the integrity of the entire protein quality control network to mitigate disease (Figure 1A).

### 3.3. Human Amylin Accumulation in the Neurovascular System: A Possible Origin of Metabolic Dementia

Emerging imaging and biomarker studies in humans are shedding light on how T2DM and insulin resistance may influence neurodegeneration [51]. Here, we consider key mechanisms connecting T2DM and AD, as suggested by in vitro and in vivo studies.

**Figure 2:**
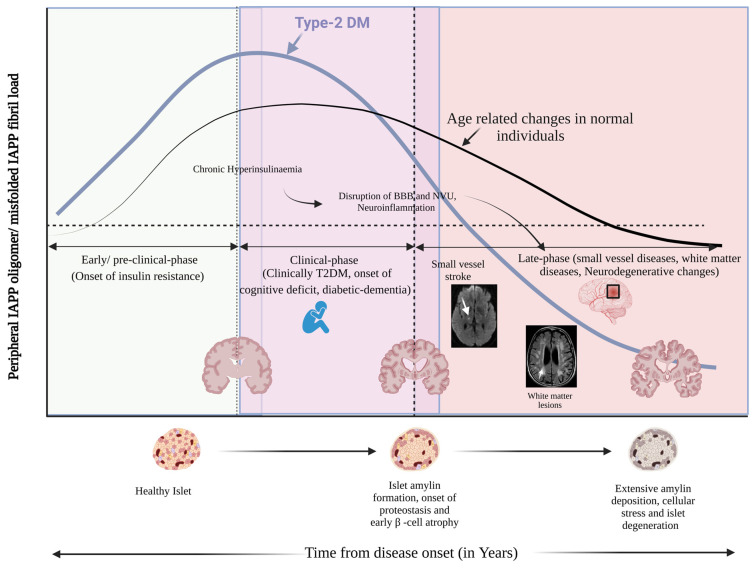
Temporal Progression of Peripheral Human Amylin Burden and Its Neurovascular Impact Across the Clinical Spectrum of Type 2 Diabetes Mellitus (T2DM). This schematic illustrates the progressive accumulation of human amylin (also known as islet amyloid polypeptide [IAPP]) oligomers and fibrils, beginning in the early stages of insulin resistance and extending through advanced neurovascular and neurodegenerative pathology associated with T2DM. The timeline is divided into three clinical phases: 1. Early Preclinical Phase (Onset of Insulin Resistance): Chronic hyperinsulinemia drives excessive human amylin secretion, initiating early islet amyloid formation even before the clinical onset of T2DM [3,6,7]. As β-cell stress accumulates, proteostatic dysfunction leads to intracellular and extracellular amylin aggregation, triggering inflammatory responses and cellular degeneration [4,25,32]. 2. Clinical Phase (Overt T2DM, Onset of Cognitive Deficits, and Diabetic Dementia): Circulating amylin oligomers cross the blood-brain barrier (BBB) and disrupt the neurovascular unit (NVU), promoting neuroinflammation, endothelial injury, and cerebral small vessel disease [52,53,54,55]. 3. Late Phase (Small Vessel Disease, White Matter Lesions, and Neurodegeneration): The pathological cascade advances to radiologically evident small vessel infarcts, white matter lesions, and progressive neurodegeneration, including diabetic dementia [20,53,56].

Multiple clinical and animal studies suggest that pancreatic amyloid (amylin) may mediate neuronal injury in AD, implicating amylin as a potential link between T2DM and dementia [52,53,54,55]. For instance, Fawver et al. [54] identified amylin in the cerebrospinal fluid and brains of patients with AD or vascular dementia, including individuals without T2DM. Postmortem analyses revealed co-localized deposits of amylin and Aβ in brain tissue from diabetic patients with vascular dementia or AD, as well as in non-diabetic AD patients [53,54]. Jackson et al. [53] reported that patients with chronic T2DM exhibited amylin deposits in the temporal lobe gray matter, often co-depositing with Aβ. Strikingly, a significant amylin deposition was also found in the cerebral blood vessels and parenchyma of patients with late-onset AD who had no clinical history of T2DM [53] (Figure 2). These findings suggest that amylin can traverse the blood-brain barrier and exacerbate amyloid pathology in the brain.

Animal models strongly support amylin’s neurodegenerative role. Transgenic rats overexpressing pancreatic amylin develop progressive neurological deficits associated with extensive amylin accumulation in the brain [56]. Compared to wild-type rats, these amylin-overexpressing rats showed large amylin deposits (>50 μm) and elevated oligomeric amylin levels in the brain. More recently, Oskarsson et al. [52] demonstrated that intravenous injection of preformed amylin fibrils (and Aβ fibrils) into transgenic mice expressing human amylin-induced amyloid deposition in both the pancreas and the brain. This in vivo “cross-seeding” supports the idea of a molecular link between peripheral amylin and cerebral amyloid (Figure 2).

The bold blue curve represents the increasing peripheral burden of misfolded amylin (IAPP) in individuals with T2DM, peaking during the clinical phase and persisting in late-stage disease. In contrast, the thinner black curve reflects age-associated amyloid burden in non-diabetic individuals, which remains comparatively low. Histological illustrations and brain MRI scans at the bottom of the panel depict the sequential transitions from healthy pancreatic islets (left) to early islet amylin deposition and β-cell stress (center), to extensive islet degeneration and cerebral white matter pathology (right) [17,37,54].

### 3.4. Biophysical Links Between Pancreatic Amylin and Cerebral Amyloid-β

Multiple lines of evidence suggest mechanistic connections between pancreatic and cerebral amyloids, specifically amylin and Aβ. Amylin shares several biophysical and physiological properties with Aβ. Despite little sequence homology, the two peptides are similar in size and adopt comparable β-sheet secondary structures [57,58].

Experiments indicate that amylin can directly interact with Aβ, potentially seeding Aβ aggregation [54]. It has been hypothesized that amylin modulates Aβ conformation and aggregation, forming stable amylin–Aβ heterocomplexes [54,59,60,61]. Whether these heterocomplexes have altered structures that confer greater toxicity remains to be determined.

Aging is accompanied by an increased accumulation of amyloidogenic proteins and a decline in proteolytic clearance via autophagy-lysosome and ubiquitin-proteasome pathways. The long-term health of cells—especially terminally differentiated neurons—relies on robust protein quality control. [62] Extensive research over the past few decades has linked amyloid aggregates to a variety of cellular dysfunctions, including mitochondrial impairment [63,64,65,66], oxidative stress [63,67], aberrant receptor-mediated signaling [67,68,69,70,71,72], disruption Ca^2+^ mediated homeostasis [73,74], membrane destabilization [75,76], and microglial activation [77]. Fibrillar amyloid plaques are pathological hallmarks of both AD and T2DM, often accompanied by cellular injury and inflammation. Interestingly, the amyloid plaque burden correlates poorly with clinical symptoms; instead, smaller soluble oligomers are now thought to be the primary toxic species driving disease progression [78]. Across various amyloidosis (including Huntington’s, Parkinson’s, and prion diseases), soluble protein oligomers are better predictors of cell death and dysfunction than insoluble fibrils [79,80]. Notably, oligomeric and fibrillar aggregates may form via distinct mechanisms influenced by external cofactors (binding partners such as other proteins, metals, and lipids, among others) [81,82].

The neurotoxicity of amylin has not been as extensively characterized as that of Aβ. Nonetheless, like Aβ, human amylin is neurotoxic to cultured neurons in a dose-dependent manner, whereas non-amyloidogenic rodent amylin lacks such toxicity [83]. Furthermore, both amylin and Aβ induce mitochondrial dysfunction [84]: they reduce the activity of electron transport chain complex IV, impair cellular respiration, and increase reactive oxygen species generation [85,86]. It remains to be seen whether oligomeric vs. fibrillar forms of amylin and Aβ differ in their toxic effects on neurons [87]. However, current evidence suggests these peptides may converge on common pathways—such as mitochondrial injury and oxidative stress—to induce neurodegeneration. Further comparative studies are needed to fully elucidate these shared mechanisms of toxicity (Figure 2).

## 4. Conclusions and Future Directions

Amylin is increasingly recognized as a molecular bridge between metabolic dysfunction and neurodegeneration. While it serves essential physiological roles in glucose homeostasis, its misfolding and aggregation can drive β-cell toxicity and contribute to the progression of T2DM. Beyond the pancreas, growing evidence suggests that amylin accumulates in the brain, potentially exacerbating AD pathology through its structural and functional similarity to Aβ.

This review has outlined the mechanisms through which amylin aggregation disrupts cellular proteostasis, involving both the ubiquitin–proteasome system and the autophagy–lysosome system. Dysregulation of these protein quality control pathways amplifies amylin’s cytotoxic effects, leading to endoplasmic reticulum stress, mitochondrial dysfunction, BAX/Bcl-2–mediated apoptosis [37], and activation of inflammatory pathways such as the NLRP3 inflammasome [33,36]. These pathological processes not only compromise β-cell survival but may also extend to neurovascular and neuronal compartments, particularly under chronic metabolic stress.

Although the association between T2DM and increased dementia risk is well-established epidemiologically, direct mechanistic links involving amylin remain under investigation. The interaction between amylin and Aβ—particularly their potential to form toxic heterocomplexes—offers a compelling hypothesis for shared neurodegenerative cascades. However, several key gaps persist. Much of the current understanding is derived from in vitro studies and transgenic animal models, and definitive in vivo evidence of amylin deposition and toxicity in the human brain—especially in early disease stages—remains limited. Moreover, current neuroimaging tools and biomarker platforms are not yet optimized for detecting amylin aggregates in the central nervous system. Disentangling the specific contributions of amylin from co-occurring amyloid species such as Aβ remains a critical challenge in mixed-pathology cases.

Future investigations should prioritize the development of reliable biomarkers for central amylin accumulation and the design of imaging modalities capable of visualizing amylin pathology in vivo. Longitudinal studies in humans are essential to determine whether amylin pathology precedes or parallels cognitive decline in diabetic individuals. Mechanistic research should also explore how proteostasis regulators—such as molecular chaperones, autophagic flux modulators, and deubiquitinating enzymes—might be targeted to mitigate amylin-induced toxicity. Importantly, identifying early intervention points in the amylin aggregation cascade may help stratify patients with T2DM who are at increased risk of neurodegeneration.

In summary, preserving a balanced network of amylin synthesis, folding, and clearance is essential for maintaining both metabolic and neurological integrity. As the global burden of T2DM and dementia continues to rise, deeper insight into amylin’s dual role in peripheral and central pathology may uncover novel, integrated therapeutic strategies. Targeting amylin itself may represent a viable approach to preventing or delaying neurodegenerative progression in individuals with T2DM.
